# Supporting energy system modelling in developing countries: Techno-economic energy dataset for open modelling of decarbonization pathways in Colombia

**DOI:** 10.1016/j.dib.2023.109268

**Published:** 2023-05-26

**Authors:** F.A. Plazas-Niño, N.R. Ortiz-Pimiento, J. Quirós-Tortós

**Affiliations:** a Industrial and Business Studies School, Universidad Industrial de Santander, Bucaramanga, Colombia; b School of Electrical Engineering, University of Costa Rica, San José, Costa Rica

**Keywords:** Energy modelling, Energy transition, Energy planning, OSeMOSYS, Energy policy

## Abstract

Decarbonization pathways have emerged as a pivotal element of global climate change mitigation strategies. Energy system modelling is widely recognized as a tool to support the design of informed energy decarbonization policies. However, the development of energy models heavily relies on high-quality input data, which may pose significant challenges in developing countries where data accessibility is limited, incomplete, outdated, or inadequate. Moreover, while models may exist in countries, these are not publicly available; therefore, details cannot be retrieved, repeated, reconstructed, interoperable or auditable (U4RIA*). This paper presents an open techno-economic energy dataset for Colombia that is U4RIA-compliant as it can be used transparently to model decarbonization pathways and support energy planning in the country. Despite being country-specific, most of the data is technology-based and thus applicable to other countries. Diverse sources, assumptions, and modelling guidelines are described to facilitate the creation of new datasets. The dataset enhances the availability of energy data for policymakers, stakeholders, and researchers, not only in Colombia but also in other developing countries.

**Specifications Table**

**Table:** 

Subject	Energy
Specific subject area	Energy System Modelling
Type of data	Table
How the data were acquired	Literature survey (databases, reports from international organizations, reports from national institutions, peer-reviewed journal articles)
Data format	Raw Processed
Description of data collected	Data were collected from websites, reports, and databases of international organisations and national entities, as well as from academic articles.
Data source location	Raw data sources are listed in the different sections of this article
Data accessibility	With this article and in a repository Repository name: Mendeley Data - Techno-Economic Energy Dataset for Open Modelling of Decarbonization Pathways in Colombia Data identification number: 10.17632/wmh4kz59wz.1 Direct URL to data: https://data.mendeley.com/datasets/wmh4kz59wz/1

## Value of the Data


 

• This dataset can be utilized to develop energy system models and assess decarbonization pathways for Colombia. Depending on the design of the modelling process, other policy insights can also be obtained.
• The dataset covers the entire chain of the energy system in Colombia, which is inexistent in the current literature.
• Analysts, policymakers, and the scientific community can employ the dataset and the methods described for conducting energy studies not only in Colombia but also in countries with similar characteristics.
• The design of this dataset can serve as a benchmark for similar studies in energy system modelling, promoting the adoption of open data and transparency.

## 1 Objective

The provision of this open dataset is expected to promote greater transparency, collaboration, and knowledge sharing among the research and modelling communities, thereby advancing the state-of-the-art in energy modelling and contributing to more effective policymaking. This effort is in line with the U4RIA goals [1], which encompass Ubuntu, Retrievability, Reusability, Repeatability, Reconstructability, Interoperability, and Auditability. Furthermore, this dataset can serve as an archetype for future energy system modelling assessments in developing countries.

## 2 Data Description

**Fig. 1:**
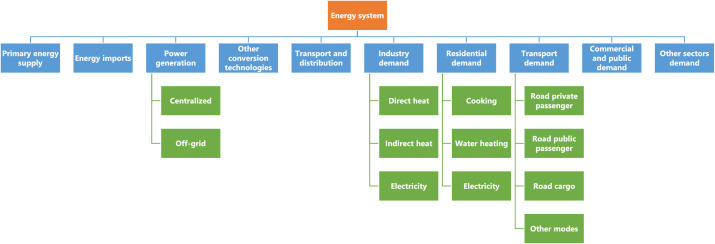
Categorization of technology data.

The data provided in this paper were gathered for the assessment of decarbonization pathways in Colombia using the OSeMOSYS framework [2]. However, the data available through this document are independent of the tool. The dataset presented was collected from websites, reports, and databases of international organizations and national entities, as well as from academic articles. It includes historical and/or projected data of end-use demands, capital and operating costs, efficiencies, operational lifetimes, capacity factors, residual capacities, emission factors, and energy availabilities. The dataset has been made openly accessible in the Mendeley Data Repository and can be accessed via the following link: https://data.mendeley.com/datasets/wmh4kz59wz/1. For better understanding, technologies have been divided into 10 categories, covering the entire chain of the energy system from primary energy supply to end-use demands (see Fig. 1). The complete list of technologies is available in the repository in the Excel file SETS, under the sheet TECHNOLOGY.

### 2.1 Demands

The modelling included 37 end-use demands in different sectors. For instance, we represented energy demand for cooking services in the residential sector and energy demand for public passenger transport by taxi. End-use demands for 2021 were obtained from the national useful energy balance [3]. The projected demand data were calculated based on the expected growth of the gross domestic product (GDP). Table 1 shows an excerpt of the end-use demand data for key years. The complete end-use demand data are available in the repository in the Excel file MODEL DATA, under the sheet DEMAND.

**Table 1:** End-use demand for key years (excerpt).

Category/Unit	End-use demand	2021	2030	2040	2050
Industry demand (PJ)	Direct heat	72.2	100.3	137.4	188.3
	Indirect heat	89.4	124.3	170.3	233.3
	Electricity	27.6	38.4	52.6	72.1
Residential demand (PJ)	Cooking	37.2	51.7	70.8	97.0
	Refrigeration	5.4	7.5	10.2	14.0
	Lightening	0.8	1.2	1.6	2.2
Transport demand (Gpkm)	Light duty vehicle transport	32.9	45.7	62.6	85.8
	Four-wheel drive transport	9.5	13.1	18.0	24.7
	Motorcycle transport	87.1	121.0	165.8	227.2
	Taxi transport	27.3	37.9	52.0	71.2
	Microbus transport	148.8	206.8	283.4	388.3
	Bus transport	341.9	475.1	650.9	891.9

### 2.2 Capital Costs

The capital cost data represent overnight costs from 2021 to 2050 for different technologies. Projected data of capital costs were considered when available, otherwise constant values were assumed. Table 2 shows an excerpt of the capital cost data for selected technologies and key years. The complete capital cost data are available in the repository in the Excel file MODEL DATA, under the sheet CAPITAL COST.

**Table 2:** Capital cost for key years (excerpt).

Sector/Unit	Technology	2021	2030	2040	2050
Power generation (MUSD/PJ/year)	Coal power plant	81.4	69.7	63.9	59.3
	Coal power plant + CCS	146.8	120.0	99.2	90.5
	Natural gas power plant	30.0	26.4	25.2	24.2
	Natural gas power plant + CCS	72.6	57.9	50.0	45.8
Other conversion technologies (MUSD/PJ/year)	Steam Methane Reforming plant	20.8	19.5	18.3	17.1
	Steam Methane Reforming plant + CCS	34.1	32.9	31.6	30.4
	ALK Electrolyzer	42.9	34.1	26.5	20.0
	PEM Electrolizer	60.7	47.2	35.7	26.2
Residential demand (MUSD/PJ/year)	Wood stove	11.6	11.6	11.6	11.6
	Natural gas stove	5.5	5.5	5.5	5.5
	Electrical stove	12.1	12.1	12.1	12.1
	LPG stove	3.6	3.6	3.6	3.6
Transport demand (MUSD/Gpkm/year)	Gasoline bus	6.9	6.9	6.9	6.9
	Diesel bus	5.7	5.7	5.7	5.7
	Electric bus	67.7	48.9	28.0	7.1
	Hydrogen bus	97.2	97.2	52.1	6.9

### 2.3 Fixed Costs

Fixed costs represent operational and maintenance costs that are independent of the activity of technologies. Projected data of fixed costs were considered when available, otherwise constant values were assumed. Table 3 presents an excerpt of the fixed cost data for selected technologies and key years. The complete fixed cost data are available in the repository in the Excel file MODEL DATA, under the sheet FIXED COST.

**Table 3:** Fixed cost for key years (excerpt).

Sector/Unit	Technology	2021	2030	2040	2050
Power generation (MUSD/PJ/year)	Solar PV Utility	0.65	0.48	0.45	0.42
	Solar PV utility + battery system	1.15	0.74	0.69	0.64
	Onshore wind power plant	1.35	1.24	1.14	1.05
	Offshore wind power plant	3.01	2.46	2.19	2.02
Transport and distribution (MUSD/PJ/year)	Recharging station	1.34	1.12	0.92	0.75
	H_2_ Refuelling station	0.16	0.16	0.16	0.16
	Bio-blended gasoline transport and distribution	0.03	0.03	0.03	0.03
	Bio-blended diesel transport and distribution	0.03	0.03	0.03	0.03
Industry demand (MUSD/PJ/year)	Natural gas boiler	0.04	0.04	0.04	0.04
	Natural gas boiler + CCS	1.22	1.12	1.06	1.06
	Electricity boiler	0.19	0.19	0.19	0.19
	Hydrogen boiler	0.18	0.18	0.18	0.18
Transport demand (MUSD/Gtkm/year)	Gasoline truck	8.02	8.02	8.02	8.02
	Diesel truck	4.15	4.15	4.15	4.15
	Electricity truck	13.22	13.22	8.69	4.16
	Hydrogen truck	45.95	45.95	25.15	4.35

### 2.4 Variable Costs

Variable costs represent the fuel costs in the case of primary energy supply and import technologies, and the variable non-fuel costs for the rest of the technology categories. The crude oil extraction cost included the cost of transport to the refinery [4]. The costs of imports were gathered from estimations performed by the Unit of Mining and Energy Planning (UPME [5]). Table 4 presents an excerpt of the variable cost data for selected technologies and key years. The complete variable cost data are available in the repository in the Excel file MODEL DATA, under the sheet VARIABLE COST.

**Table 4:** Variable cost for key years (excerpt).

Sector/Unit	Technology	2021	2030	2040	2050
Primary energy supply (MUSD/PJ)	Coal extraction	2.84	2.84	2.84	2.84
	Crude oil extraction	1.57	1.57	1.57	1.57
	Natural gas extraction	4.20	4.20	4.20	4.20
	Bagasse production	2.55	2.55	2.55	2.55
Energy imports (MUSD/PJ)	Diesel imports	8.67	8.66	10.62	12.79
	Gasoline imports	10.09	11.04	14.93	19.14
	Jet Fuel/Kerosene imports	13.98	23.39	31.15	39.62
	Fuel oil imports	8.43	13.27	17.07	21.23
Power generation (MUSD/PJ)	Coal power plant	2.22	1.94	1.94	1.94
	Coal power plant + CCS	4.17	3.89	3.61	3.61
	Natural gas power plant	0.56	0.56	0.56	0.56
	Natural gas power plant + CCS	1.67	1.67	1.67	1.67
Industry demand (MUSD/PJ)	Biomass furnace	0.47	0.47	0.47	0.47
	Biomass furnace + CCS	2.00	2.00	2.00	2.00
	Coal furnace	0.47	0.47	0.47	0.47
	Coal furnace + CCS	2.41	2.41	2.14	2.14

### 2.5 Emissions Factors

We considered equivalent emission factors that include carbon dioxide (CO_2_), methane (CH_4_), and nitrous oxide (NO_2_). The emission factor data were obtained from [6], which summarize emission data from the Intergovernmental Panel on Climate Change (IPCC) and domestic data of national studies. Table 5 presents the consolidated data used for emission factor calculations as described in Section 3.5. For CCS technologies, an efficiency of 90% in capturing CO_2_ emissions is assumed [7], and emission factors by technology are recalculated accordingly. The limit of geological storage for CO_2_ is estimated at 360 MtCO_2_ considering the potential of CO_2_ injection for enhanced recovery in oil and gas reservoirs [8]. The complete emission factor data are available in the repository in the Excel file MODEL DATA, under the sheet EMISSION FACTOR.

**Table 5:** Fuel emission factors.

Fuel	Emission factor (kg/TJ)			Equivalent emission factor (ktCO_2_e/PJ)
	CO_2_	CH_4_	N_2_O	
Coal	88136	10	1.5	88.8
Fuel oil	80460.3	3	0.6	80.7
Diesel	74233.4	3.9	3.9	75.4
Natural gas	55539.11	92	3	58.9
Gasoline	69323.7	33	3.2	71.1
LPG	67185.1	5	0.1	67.4
Jet fuel-Kerosene	73939.6	10	2	74.7

### 2.6 Operational Lifetimes

The operational lifetime represents the standard value of a technology's lifespan in number of years. Table 6 presents an excerpt of the operational lifetime data for selected technologies. For technologies with no capital or fixed costs, a default lifetime of 100 years is assigned. The complete operational lifetime data are available in the repository in the Excel file MODEL DATA, under the sheet LIFETIME.

**Table 6:** Technology operational lifetimes (excerpt).

Sector	Technology	Lifetime (years)
Other conversion technologies	Refinery	50
	LPG plant	30
	Destillery	25
	Biodiesel plant	25
Transport and distribution	Transmission electricity technology	60
	Distribution electricity technology	60
	Recharging station	30
	H2 Refuelling station	30
Transport demand	Gasoline motorcycle	20
	Diesel motorcycle	20
	Natural gas motorcycle	20
	Electricity motorcycle	17
Commercial and public demand	Hydrogen motorcycle	17
	Natural gas boiler	25
	Electricity boiler	25
	Low efficiency refrigerator	15

### 2.7 Efficiencies

Efficiency in the modelling process represents the ratio between output energy and input energy. Efficiency data is collected from multiple sources, as described in Section 3.7. Due to uncertainty, efficiencies were assumed to be constant. When data was unavailable, efficiency was assumed to be equal to 1. Table 7 shows an excerpt of the efficiency data for some technologies. All technology efficiencies are expressed as a percentage, except for transport technologies, whose efficiency is expressed in Gpkm/PJ for passenger transport and Gtkm/PJ for cargo transport. In the case of crude oil refining, the output ratios for the different petroleum derivatives are estimated from [9]. Table 8 presents the output ratios for the refinery technology. The complete efficiency data is available in the repository in the Excel file MODEL DATA, under the sheet EFFICIENCY.

**Table 7:** Technology efficiencies (excerpt).

Sector	Technology	Efficiency
Power generation (%)	Natural gas power plant	42
	Natural gas power plant + CCS	38
	Biomass power plant	25
	Biomass power plant + CCS	20
Industry demand (%)	Natural gas boiler	79
	Natural gas boiler + CCS	75
	Electricity boiler	89
	Hydrogen boiler	82
Residential demand (%)	Wood stove	4
	Natural gas stove	41
	Electrical stove	65
	LPG stove	43
Transport demand (Gpkm/PJ)	Gasoline light duty vehicle	0.49
	Diesel light duty vehicle	0.53
	Electricity light duty vehicle (BEV)	2.78
	Hydrogen light duty vehicle (FCEV)	1.21

**Table 8:** Efficiency for refinery technology.

Energy carrier	Output ratio
Gasoline	0.2268
Diesel	0.3854
LPG	0.0181
Jet fuel/kerosene	0.0592
Fuel oil	0.1319

### 2.8 Capacity Factors

Capacity factor is defined as the ratio of energy produced by a generating unit for the period considered to the energy that could have been produced at continuous full operation during the same period. For power generation and other conversion technologies, the capacity factors were reported using different sources and considerations as depicted in Section 3.8. Table 9 presents an excerpt of the capacity factor data for power generation technologies. For demand technologies, we assumed that installed capacity is fully available, and capacity factor is equal to 1. The complete capacity factor data is available in the repository in the Excel file MODEL DATA, under the sheet CAPACITY FACTOR.

**Table 9:** Capacity factors for power generation technologies.

Technology	Capacity factor
Coal power plant	0.85
Coal power plant + CCS	0.85
Natural gas power plant	0.85
Natural gas power plant + CCS	0.85
Biomass power plant (wood)	0.66
Biomass power plant + CCS (wood)	0.80
Hydro power plant (dam)	0.60
Hydro power plant (run-of-river)	0.41
Geothermal power plant	0.80
Nuclear power plant (SMR)	0.94
Solar PV Utility	0.21
Solar PV utility + battery system	0.26
CSP power plant	0.58
Onshore wind power plant	0.34
Offshore wind power plant	0.44
Distributed PV network	0.24
Individual photovoltaic solution (Rooftop PV)	0.12
Diesel plant standalone	0.40

### 2.9 Residual Capacities

Residual capacity represents the installed capacity of a technology each year. Projected data of power generation included the planned power plants from 2022 to 2026, as detailed in the repository's Excel file SUPPLEMENTARY DATA, under the sheet ADDITIONAL PP. Other installed capacities for conversion technologies were assumed to remain constant until 2050. On the demand side, installed capacities were decreased linearly based on the technology's operational life. Table 10 presents an excerpt of the residual capacity data for selected technologies. The complete residual capacity data is available in the repository's Excel file MODEL DATA, under the sheet RESIDUAL CAPACITY.

**Table 10:** Technology residual capacities for key years (excerpt).

Sector/Unit	Technology	2021	2030	2040	2050
Power generation (PJ/year)	Solar PV Utility	4.26	37.43	37.43	37.43
	Hydro power plant (dam)	326.08	401.77	401.77	401.77
	Onshore wind power plant	0.58	65.92	65.92	65.92
	Natural gas power plant	82.62	92.78	92.78	92.78
Industry demand (PJ/year)	Biomass furnace	1.75	1.40	0.70	0.00
	Coal furnace	21.22	16.97	8.49	0.00
	Natural gas furnace	39.62	31.69	15.85	0.00
	Electricity furnace	10.70	8.03	2.68	0.00
Residential demand (PJ/year)	Wood stove	3.68	1.94	0.00	0.00
	Natural gas stove	22.85	12.03	0.00	0.00
	Electrical stove	1.00	0.53	0.00	0.00
	LPG stove	9.65	5.93	0.00	0.00
Transport demand (Gpkm/year)	Gasoline bus	74.66	41.07	3.73	0.00
	Diesel bus	245.34	134.94	12.27	0.00
	Natural gas bus	16.54	9.10	0.83	0.00
	Electricity bus	5.34	2.51	0.00	0.00

### 2.10 Annual Potentials and Reserves

We have considered fossil fuel, nuclear, biomass, and renewable energy as categories of primary energy supply. Fossil fuel reserves were reported in volume and mass quantities according to official government data. Potential reserves of uranium for nuclear energy production were also included. The estimated potential of renewable energy technologies was derived from technical studies. Annual biomass production was estimated based on assumptions described in Section 3.10. Table 11 provides a description of the availability of primary energy resources by category. The data is also available in the repository's Excel file MODEL DATA, under the sheet RESERVES-POTENTIALS.

**Table 11:** Energy availability per source.

Category	Resource	Unit	Availability	Reference
Fossil fuels	Crude oil^a^	Mbl	5704	[10]
	Natural gas^b^	Tcf	10.9	[11]
	Coal	Mt	1586	[12]
Nuclear	Uranium	kt	11	[13]
Renewable energy	Hydro (dam)	GW	51.2	[14]
	Hydro (run-of-river)	GW	27.8	
	Solar PV	GW	8172.2	[15]
	Solar CSP^c^	GW	17	Estimated
	Onshore wind	GW	35.2	[15]
	Offshore wind	GW	50	[16]
	Geothermal	GW	1.2	[17]
Biomass	Sugarcane (for bioetanol)	PJ/year	573	[14]
	Oilpalm (for biodiesel)	PJ/year	315.5	
	Bagasse	PJ/year	166.5	
	Firewood	PJ/year	260	
	Agriculture and forestal residues	PJ/year	165	

a Intermediate scenario is used to consider future additions of crude oil reserves based on historical exploration success rates

b Intermediate scenario is used to consider future additions of natural gas reserves based on historical exploration success rates

c There is no available data about the potential for CSP in Colombia, whereby an estimation of 17 GW is assumed considering the same installed power plant capacity in 2021.

## 3 Experimental Design, Materials, and Methods

The dataset was compiled through a comprehensive literature review. Data was gathered from websites, reports, and other databases of international organizations and national entities, as well as from academic articles. The raw data was organized, analyzed, processed, and standardized according to the requirements of the modelling. We provide detailed information on the data sources, assumptions, and processing methods implemented in the construction of the dataset in the following sections.

## 3.1 Demands

We obtained the end-use demands for 2021 from the national useful energy balance [3]. In the case of the transport sector, the end-use demand was estimated using data on consumed energy per vehicle type from [3], and vehicle efficiency from [18,19], as shown in Eq. (1). The projected demand data were calculated by multiplying the baseline 2021 demands by the expected percentage increase in GDP. For 2022, the percentage increase was set at 8% due to the rebound from the COVID-19 pandemic [20]. For the period 2023-2050, the rate was estimated at 3.2% per year based on the average GDP observed during the period 2012-2019 [21].

(1) $$Technology\;end-use\;demand\;=Consumed\;energy\;\left[PJ\right]*Technology\;efficiency\left[\frac{Gpkm}{PJ}\right]$$

## 3.2 Capital Costs

Capital cost data were collected from various sources, as summarized in Table 12. The projected costs were taken directly from the literature sources and were not calculated. All costs were converted to 2021 USD using the average euro-dollar exchange rate from [22] and the inflation rate based on [23].

**Table 12:** List of sources for capital cost data.

Category	References
Power generation	[24], [25], [26], [27]
Other conversion technologies	[28], [29], [30]
Transport and distribution	[27,30] and estimations described below
Industry demand	[27,^30^,31]
Residential demand	Estimations based on commercial prices in the Colombian market
Transport demand	[29,^32^,33]
Commercial and public demand	[30] and estimations based on commercial prices in the Colombian market

We addressed the lack of data by making the following estimations:

A. Transportation and distribution of fossil fuels were represented by single technologies to capture the capacity and cost of expansion. To account for the lack of information and to avoid the requirements of locations and distances, a method to quantify the cost per unit of capacity was implemented based on [34]. We multiplied the variable cost of transport and distribution by the total annual demand of the energy carrier, and then divided it by the total installed capacity for the reference year 2021. Eq. (2) shows the calculation for this process, and Table 13 summarizes the estimations performed.
  (2) $$Transport-distribution\;capital\;cost\left[\frac{USD}{MW}\right]=\frac{\left(Variable\;transport-distribution\;cost\;\left[\frac{USD}{MWh}\right]*Annual\;demand\;\left[MWh\right]\right)}{Installed\;capacity\;\left[MW\right]}$$

**Table 13:** Transportation and distribution technologies cost.

Energy carrier	Transport-Distribution cost (USD/MWh)	Annual demand (MWh) [9]	Network capacity (MW)	Transport-Distribution capital cost (USD/kW)
Coal	1.48 [35]	22447000	2562.45 [9]	12.96
Gasoline	10.21 [38]	79339000	26778.89 [36]	30.25
Diesel	9.6 [38]	87128000	28485.52 [36]	29.36
Liquified petroleum gas (LPG)	14.91 [39]	10014000	5043.08 [37]	29.61
Natural gas	23.7 [40]	55316000	45208.33 [11]	29
B. Capital costs of transport technologies were converted from USD per vehicle to USD per passenger-kilometre or USD per tonne-kilometre. We divided the unit cost of each vehicle type [32] by the product of the activity factor and the occupancy factor [18]. Eq. (3) shows the structure of this calculation. For battery electric vehicles (BEV), plug-in hybrid electric vehicles (PHEV), and fuel cell electric vehicles (FCEV), a conservative approach was assumed, considering cost parity with internal combustion engine (ICE) technologies by 2050 based on [32]. Complete data on transport costs are available at the repository in the Excel file SUPPLEMENTARY DATA in the sheet TRANSPORT COST.
  (3) $$\begin{array}{ccc} & & Transport\;technology\;cost\left[\frac{USD}{pkm/year}\right]\\ & & \;=\frac{Unit\;technology\;cost\;\left[\frac{USD}{veh}\right]}{Activity\;factor\;\left[\frac{km}{year}\right]*Occupancy\;factor\;\left[\frac{p}{veh}\right]} \end{array}$$
C. The capital costs of residential technologies were estimated using the commercial prices on the webpages of the major retail companies in Colombia. Low efficiency appliances were categorized as C, D, E, F, and G according to the Technical Labelling Regulation (RETIQ, Spanish abbreviation), which is the national regulation that certifies the efficiency level of equipment in Colombia. High efficiency appliances belong to categories A and B according to the RETIQ. Eq. (4) presents the calculation to estimate the cost per unit of capacity. Complete data on residential costs are available at the repository in the Excel file SUPPLEMENTARY DATA in the sheet RESIDENTIAL COST.
  (4) $$Residential\;technology\;cost\left[\frac{USD}{kW}\right]=\frac{Unit\;technology\;cost\;\left[USD\right]}{Capacity\;\left[kW\right]}$$
D. Due to the lack of available data for furnaces and boilers coupled with CCS technologies in the industry sector, capital costs were estimated using the differential costs in power plants. For instance, if the capital cost of a coal power plant is 2400 USD/kW, and the capital cost of a coal power plant with CCS is 4600 USD/kW, then the differential cost is 2200 USD/kW. This differential cost is added to the capital cost of a coal furnace and a coal boiler to represent the coal technologies with CCS in the industry sector.

## 3.3 Fixed Costs

Fixed cost data were gathered from several sources, as summarized in Table 14. The projection of costs was taken from the same literature sources and was not calculated. All costs were converted to 2021 USD using the average euro-dollar exchange rate from [22] and the inflation rate based on [23].

**Table 14:** List of sources for fixed cost data.

Category	References
Power generation	[24], [25], [26], [27]
Other conversion technologies	[28], [29], [30]
Transport and distribution	[30,41] and estimations described below
Industry demand	[27,^30^,31]
Transport demand	Estimations based on [14]
Commercial and public demand	[30]

In transport and distribution technologies, we assumed a fixed cost of 3% of the capital cost annually. For ICE and FCEV passenger vehicles, a fixed cost of 3% of the capital cost was considered, while for BEV and PHEV, it was assumed to be 1%, except for buses and microbuses. For buses, microbuses, and freight transport, a fixed cost of 2% of the capital cost was used for all technologies. These assumptions were based on [14]. For other transport modes, we assumed a fixed cost of 3% of the capital cost. For furnaces and boilers coupled with CCS technologies in the industry sector, we applied the same consideration described previously, and fixed costs were estimated using the differential costs in analogous power plants.

## 3.4 Variable Costs

Variable cost data were gathered from several sources, as summarized in Table 15. The projected data of fossil fuel imports were obtained from an assessment by UPME for the period between 2021 and 2037, under the reference scenario [5]. For the period 2038-2050, the data was extrapolated using a linear trend. Domestic production costs of primary energy resources were assumed to be constant. We also included a variable cost of transport and storage of CO_2_ equal to 36.1 US$/t [7], added to CCS technologies to represent the financial cost of CO_2_ infrastructure. For furnaces and boilers coupled with CCS technologies in the industry sector, we applied the same consideration described previously, and variable costs were estimated using the differential costs in analogous power plants. All costs were converted to 2021 USD using the average euro-dollar exchange rate from [22] and the inflation rate based on [23].

**Table 15:** List of sources for variable cost data.

Category	References
Primary energy supply	[4,^14^,^29^,^35^,42]
Energy imports	[5]
Power generation	[24], [25], [26]
Other conversion technologies	[27,29]
Industry demand	[27,^30^,31]
Commercial and public demand	[30]

## 3.5 Emissions Factors

The emission factor data were gathered from [6], and the calculations for finding the equivalent emission factors in terms of CO_2_e were conducted using the global warming potentials (GWP) described by [43]. Eq. (5) shows the structure of the calculation mentioned. For the modelling approach, emissions were calculated as a product of the technology activity level and the emission factor, thus the emission factor by technology will depend on the efficiency of the technology. Eq. (6) presents the basic calculation to find the emission factor by technology. Biomass resources were considered carbon neutral [44], and bioenergy technologies coupled with CCS (BECCS) were allocated the respective negative emissions.

(5) $$\begin{array}{ccc} Equivalent\;emission\;factor\left[\frac{MtCO_{2}e}{PJ}\right] & = & CO_{2}\;emission\;factor\left[\frac{Mt}{PJ}\right]*CO_{2}\;GWP\\ & & +\;CH_{4}\;emission\;factor\left[\frac{Mt}{PJ}\right]*CH_{4}\;GWP\\ & & +\;N_{2}O\;emission\;factor\left[\frac{Mt}{PJ}\right]*N_{2}O\;GWP \end{array}$$

(6) $$Emission\;factor\;by\;technology\left[\frac{MtCO_{2}e}{PJ}\right]=\frac{Fuel\;Emission\;factor\left[\frac{MtCO_{2}e}{PJ}\right]}{Technology\;efficiency}$$

## 3.6 Operational Lifetimes

Table 16 summarizes the data sources used for gathering operational lifetime values. When data were unavailable, reasonable values were assumed based on similar technologies. The lifetimes for road transport technologies were adjusted considering the lack of regulation for maximum age and the average ages of vehicles in Colombia [45].

**Table 16:** List of sources for operational lifetime data.

Category	References
Power generation	[27,^30^,^41^,46]
Other conversion technologies	[14,^28^,29]
Transport and distribution	[27,47]
Industry demand	[27,^30^,31]
Residential demand	Estimations based on commercial prices in the Colombian market
Transport demand	[29,^32^,33]
Commercial and public demand	[30] and estimations based on commercial prices in the Colombian market

## 3.7 Efficiencies

Efficiency data were gathered from different sources as summarized in Table 17. For non-hydrogen industry technologies and cooking technologies, the efficiencies are estimated from the national useful energy balance [3]. For furnaces and boilers coupled with CCS technologies in the industry sector, efficiencies were estimated using the differential efficiency in analogous power plants. In transport and distribution technologies of hydrogen and fossil fuels, we assumed an efficiency of 1 owing to a lack of data. For blending technologies, the mix percentage is set at 10% for both bioethanol and biodiesel [48].

**Table 17:** List of sources for efficiency data.

Category	References
Power generation	[14,^24^,^27^,30]
Other conversion technologies	[9,^14^,28]
Transport and distribution	[27]
Industry demand	[3,31]
Residential demand	[3,49]
Transport demand	[14,^18^,^19^,29]
Commercial and public demand	[3,49]

## 3.8 Capacity Factors

The average annual capacity factors of solar PV and onshore wind technologies were estimated using the generation and capacity information in the period 2015-2021 [50]. The annual power generation reported is divided by the theoretical power generation assuming that the installed capacity works 100% of the time, as described by Eq. (7). The capacity factors for other power and conversion technologies were obtained from reports and literature assessments as summarized in Table 18. We assumed that end-use technologies are fully available to supply the demand and thus capacity factors are equal to 1.

(7) $$Annual\;capacity\;factor=\frac{Annual\;power\;generation\;\left[GWh\right]}{Theoretical\;power\;generation\;\left[GWh\right]}$$

**Table 18:** List of sources for capacity factor data.

Category	References
Power generation	[24,^27^,^30^,^41^,^46^,50]
Other conversion technologies	[14,^28^,29]

## 3.9 Residual Capacities

Installed capacity of power plants was collected from the market operator XM for centralized generation [51] and from the Promotion and Planning Institute for Energy Solutions (IPSE) for decentralized energy [52]. Fossil fuel processing and refining capacities were gathered from different sources, as shown in Table 19. Transport and distribution installed capacities are summarized in Table 13. Installed capacity of power transmission and distribution was obtained from [14], and installed capacity of recharging stations was estimated by considering 491 chargers with 50 kW each [53]. For end-use technologies, we estimated the residual capacities in 2021 by assuming full use of installed capacity to supply demand. Eqs. (8) and (9) present the way of calculating the technology residual capacity via the energy consumed by technology and the efficiency of the technology. In the transport sector, we can also estimate the number of vehicles by category using the activity factor and occupancy factor, as illustrated in Eq. (10).

(8) $$\begin{array}{ccc} Technology\;residual\;capacity\;\left[\frac{PJ}{year}\right] & = & Energy\;consumed\;by\;technology\;\left[\frac{PJ}{year}\right]\\ & & *Technology\;efficiency\left[\%\right] \end{array}$$

(9) $$\begin{array}{ccc} Technology\;residual\;capacity\;\left[\frac{Gpkm}{year}\right] & = & Energy\;consumed\;by\;technology\;\left[\frac{PJ}{year}\right]\\ & & *Technology\;efficiency\left[\frac{Gpkm}{PJ}\right] \end{array}$$

(10) $$Number\;of\;vehicles\;\left[veh\right]=\frac{10^{9}*Technology\;residual\;capacity\;\left[\frac{Gpkm}{year}\right]}{Activity\;factor\;\left[\frac{km}{year}\right]*Occupancy\;factor\;\left[\frac{p}{veh}\right]}$$

**Table 19:** List of sources for residual capacity data.

Category	References
Power generation	[50,52]
Other conversion technologies	[36,^37^,^54^,55]

*Eqs. (9) and (10) are the same for cargo transport technologies using units of Gtkm/year

Regarding the projected residual capacities, we included the planned power plants from the renewable energy auctions in 2019 and 2021 [56,57], the Hidroituango project, and other committed projects [58]. Phase-out power plants were not considered due to information unavailability, and other residual capacities for conversion and transport-distribution technologies were assumed constant. For end-use technologies, we used simplified mortality lines, where residual capacity decreases linearly according to the operational lifetime until reaching zero, as shown by Eq. (11). In the industry sector, we considered constant residual capacity until 2025 and then the mortality line was applied. For other demand sectors, the reduction in installed capacity started from 2022. The calculations depend on the assumption of capacity factors equal to 1 in the end-use technologies. Improved estimations of installed capacities and projections are possible if technology inventory data are available.

$$Residual\;capacity\;in\;year\;n\;=Residual\;capacity\;in\;year\;0-\frac{Residual\;capacity\;in\;year\;0}{Operational\;lifetime}*n,$$

(11) ∀n|Residualcapacity≥0

## 3.10 Annual Potentials and Reserves

Crude oil and natural gas reserves in 2021 were 2039 Mbl and 3.2 Tcf respectively [59]. We considered projected reserves for the period 2021-2050 equal to 5704 Mbl and 10.9 Tcf based on the intermediate scenarios of future availability of fossil fuels assessed by UPME [10,11]. These values are highly uncertain but are conservative when considering the historical incorporation of new fossil fuel reserves in the past 14 years [59]. Solar PV and onshore wind estimations considered regional energy potential and availability of land for power plant deployment [15]. Hydro potential was obtained from a national estimation [14]. Geothermal potential was estimated based on hot springs [17] and did not consider reconversion of oil wells to geothermal wells. The national roadmap of offshore wind energy was used to obtain data on potential installed capacity of the technology [16]. Biomass primary supply was estimated using data from [14], assuming a potential land area of 1000 kha and the present energy crop yields. Fuelwood was limited using the data available for a crop of Eucalyptus. Bagasse potential was estimated using a residue to product ratio of 0.31 with respect to sugarcane potential. Table 20 summarizes the data used in the biomass estimations.

**Table 20:** Estimated biomass potentials.

Energy crop	Energy crop yield (GJ/ha)	Land availability (kha)	Estimated potential (PJ/year)
Sugarcane	573	1000	573
Oilpalm	216	1000	216
Fuelwood	260	1000	260

## Ethics Statement

Not applicable.

## CRediT authorship contribution statement

**F.A. Plazas-Niño:** Conceptualization, Methodology, Formal analysis, Investigation, Data curation, Writing – original draft. **N.R. Ortiz-Pimiento:** Supervision, Writing – review & editing. **J. Quirós-Tortós:** Supervision, Writing – review & editing.

## Declaration of Competing Interest

The authors declare that they have no known competing financial interests or personal relationships that could have appeared to influence the work reported in this paper.

## Data Availability

Techno-Economic Energy Dataset for Open Modelling of Decarbonization Pathways in Colombia (Reference data) (Mendeley Data).
